# Infant-Directed Speech From a Multidimensional Perspective: The Interplay of Infant Birth Status, Maternal Parenting Stress, and Dyadic Co-regulation on Infant-Directed Speech Linguistic and Pragmatic Features

**DOI:** 10.3389/fpsyg.2022.804792

**Published:** 2022-05-09

**Authors:** Maria Spinelli, Francesca Lionetti, Maria Concetta Garito, Prachi E. Shah, Maria Grazia Logrieco, Silvia Ponzetti, Paola Cicioni, Susanna Di Valerio, Mirco Fasolo

**Affiliations:** ^1^Department of Neuroscience, Imaging and Clinical Sciences, G. d’Annunzio University of Chieti-Pescara, Chieti, Italy; ^2^Division of Developmental Behavioral Pediatrics, Department of Pediatrics, University of Michigan, Ann Arbor, MI, United States; ^3^Department of Maternal and Child Health, Santo Spirito Hospital, Pescara, Italy

**Keywords:** infant-directed speech, preterm birth, parenting stress, dyadic co-regulation, mother-infant interaction

## Abstract

Infant-directed speech (IDS), the particular form of spontaneous language observed in interactions between parents and their infants, is a crucial aspect of the mother-infant interaction and an index of the attunement of maternal linguistic input to her infant communicative abilities and needs during dyadic interactions. The present study aimed to explore linguistic and pragmatic features of IDS during mother-infant interactions at 3-month of infant age. The effects of infant (birth status: preterm vs. full-term birth), maternal (perceived parenting stress) and dyadic (dyadic co-regulation) factors on IDS were explored. Results evidenced few differences between the groups on IDS linguistic characteristics. Moreover, observing the interaction of birth status and dyadic co-regulation, full-term mothers varied their IDS pragmatic features according to the quality of co-regulation while preterm mothers did not. Parenting stress was associated to specific linguistic IDS features independently from the birth status. Findings are discussed underling implications for the study of preterm dyads interactions and the importance to consider the interplay of several factors in affecting the quality of IDS.

## Introduction

Infant-directed speech (IDS) is the particular form of spontaneous language observed in interactions between parents and their infants (Ferguson, 1964; Saxton, 2009; Saint-Georges et al., 2013). Compared to adult-directed speech (ADS), this type of verbal interaction is characterized by a simplification of speech demonstrated by fewer utterances, lexical and syntactic simplification, specific pragmatic functions, and emphasized prosody (see Soderstrom et al., 2008, for a review; Fernald and Simon, 1984; Genovese et al., 2020).

Characteristics of IDS and associations with infant development have been studied extensively over the last few decades. The specific vocal patterns and linguistic features of IDS are an important part of mother-infant interactions, and play a role in regulating and attracting infant attention, making linguistic input more apparent and salient to infants, and helping infant interpretation of the emotional signals of adult speakers (Saint-Georges et al., 2013; Spinelli et al., 2017). Decades of research have further elucidated the role of infant directed speech on facilitating co-regulated attention and affect during mother-child interactions, and the potential help to foster early language development (Ferguson, 1964; Soderstrom, 2007).

Patterns of child-directed speech have been observed from infancy to childhood (see for example Kitamura et al., 2001; Genovese et al., 2020), and have demonstrated patterns of stability and instability over time. Some characteristics of IDS, such as the prosodic features tend to be more stable, with minor variations over time (Kitamura and Burnham, 2003). Other features, (e.g., linguistic characteristics), appear to change over time (Bornstein et al., 1992; Ko, 2012), with mothers adjusting their speech to make it more complex and variable, in line with their infants and children’s increasing cognitive and communicative abilities (Genovese et al., 2020).

The specific features of IDS, its important role for infant and child development, its variations with infant age, make IDS a crucial aspect of the mother-infant interaction and an index of the attunement of maternal linguistic input to her infant and child communicative abilities and needs during dyadic interactions (Saint-Georges et al., 2013). While it is believed that IDS varies *within* the dyadic interaction according to the communicative needs and aims of the mother and infant communication, as well as *among* mothers according to maternal ability to attune to infant’s needs, most studies of IDS have focused on descriptive features of IDS and/or its associations with infants outcomes. The intra and inter-individual differences in IDS have been under-explored and is a current gap in the research.

The main aim of the present study is to contribute to this field of research by exploring how select individual and dyadic factors, previously associated with the quality of the mother-infant interaction, are also associated with IDS features. We examined these associations during the preverbal age of 3 months of age. This age is crucial for several reasons, first because the growing communicative abilities of the infant, i.e., vocalizations, smiles and movements, make him/her contribution to the interaction very relevant, second because at this age maternal voice is considered to be one of the main interactive modalities during dyadic face-to-face interactions, all of this resulting in moments of dyadic shared affect and attention (Stern et al., 1983; Lavelli and Fogel, 2013). We explored infant characteristics [i.e., infant’s birth condition, represented by preterm (PT) vs. full-term (FT) birth], maternal characteristics (i.e., maternal well-being, represented by mother’s levels of parenting stress), and dyadic characteristics such as the quality of dyadic co-regulation, and their associations with IDS characteristics (i.e., linguistic and pragmatic aspects).

Preterm birth–birth before the 37th week of gestational age—is a non-normative birth experience, associated with perturbations in several areas of newborn development. One area of developmental vulnerability centers on the communicative abilities of preterm infants. Compared to infants born full-term, preterm infants, especially during the first months of life, show different communicative abilities. PT infants are reported to be less reactive to social cues than FT infants, and manifest interactions characterized by lower attentional control (Bhutta et al., 2002; Clark et al., 2008), diminished alertness and responsivity (Goldberg, 1978; Goldberg and DiVitto, 1995), greater passivity, less initiation (Sajaniemi et al., 1998), increased irritability (Hughes et al., 2002; Larroque et al., 2005), and fewer expressions of positive affect (Garcia Coll et al., 1992). In addition, compared to their FT counterparts, PT infants are less interactive, and vocalize less in response to the utterances of their mother (Reissland and Stephenson, 1999).

These interactive difficulties complicate the social and affective exchanges between PT infants and their mothers, who may find the infant’s cues and reactions difficult to understand (Loi et al., 2017). There is some evidence to suggest that mothers of PT infants tend to look, smile, vocalize, affectionately touch them less often, and appear to be less competent at coordinating their social behaviors with the infant’s signals, compared to mothers of FT newborns (Forcada-Guex et al., 2006; Olafsen et al., 2006; Feldman and Eidelman, 2007; De Schuymer et al., 2011). However, there is also variability within the pattern of PT dyadic interactions, with not all mothers and PT infants showing the same interactional difficulties (Agostini et al., 2014; Sansavini et al., 2015; Neri et al., 2017). Underscoring this finding, a recent meta-analysis examining studies on maternal sensitivity of PT and FT mothers reported a general lack of evidence of group differences (Bilgin and Wolke, 2015).

Similar mixed findings have been reported by the few studies exploring differences in IDS between PT and FT mothers during dyadic interactions. Some studies found that mothers of PT infants demonstrated contingent vocalizations more frequently (Reissland and Stephenson, 1999), used more complex interrogatives (Reissland et al., 1999), and tended to interrupt silent pauses in the conversation more often (Salerni et al., 2007) than FT mothers. Other studies failed to find differences in IDS linguistic features between FT and PT dyads (i.e., mean lenght of utterance (MLU), type/token ratio, quantity of tokens and types per minute and frequency of utterances per minute (Salerni et al., 2007; Suttora et al., 2020a) and the total amount of speech (Adams et al., 2018).

It is thought that mothers of children born PT may need to modulate their interactions to the appropriate level of linguistic stimulation to avoid over- or underwhelming the PT infant’s communication and arousal regulation capacities (Suttora and Salerni, 2011). Some mothers might be more able to do that, others less able, not only because of the infant prematurity condition, but also because of specific maternal characteristics.

It is well known that the interactive difficulties of PT mothers are not homogeneous, but vary by conditions related to neonatal and maternal health. Interactional differences are attributed to both the level of neonatal risk, e.g., mothers of more at risk infants have demonstrated lower sensitivity to infants cues (Agostini et al., 2014; Bilgin and Wolke, 2015), and also to the level of maternal wellbeing. Preterm birth is a non-normative transition to motherhood characterized by the sudden interruption of pregnancy, the subsequent fear for and worry about the infant’s survival and health condition, and the experience of caregiving in the highly technological environment of the Neonatal Intensive Care Unit (NICU) for an extended time. These experiences can result in preterm mothers experiencing high levels of psychological distress (Coppola et al., 2007; Feldman and Eidelman, 2007; Spinelli et al., 2016). This heightened psychological distress might alter mothers’ perceptions and attitudes toward the infant, rendering her experience of parenting stressful and demanding. There is evidence suggesting that the majority of mothers of PT infants experience high levels of parenting stress (Brummelte et al., 2011; Gray et al., 2012), with associated negative impacts on the quality of dyadic interactions (Spinelli et al., 2013). Higher parenting stress has been associated with less attuned, less positive interactions, and more intrusive behaviors (Spinelli et al., 2013; Suttora et al., 2020b). To our knowledge, the associations between maternal emotional difficulties (e.g., parenting stress) and the quality of IDS in mothers of PT infants have not been examined, a gap in the science which this research will address.

### The Present Study

While it is well described that PT birth poses a risk for suboptimal mother-infant interactions, the impact of preterm birth on the quality of dyadic interactions and maternal wellbeing varies. As a consequence, the quality of maternal verbal communication varies. Considering the role of IDS in infant development, there is a growing need to explore the interactive effects of preterm birth with other potential sources of variability in the quality of mother-infant interactions and their associations with maternal communicative behavior in IDS.

The aim of the present study was to explore the interactive effects of PT birth, maternal parenting stress, and the quality of dyadic co-regulation on the linguistic and pragmatic features of IDS. We considered dyadic co-regulation as a form of dyadic process that consider both the infant and the mother behaviors. Co-regulation refers to a form of coordinated action between participants that involves a continuous mutual adjustment of actions and intentions (Fogel and Thelen, 1987). We expected to find variability in IDS features which was associated with PT birth and maternal psychological characteristics. Specifically, since PT mothers are more at risk for psychological difficulties, we hypothesized that PT mothers IDS would be more affected by parenting stress, with PT mothers with higher parenting stress demonstrating IDS less appropriate to infant age characterized by, i.e., low quantity and variety of verbal interaction, more control sentences. Moreover, we expected to find PT and FT mothers using different patterns of IDS during moments of shared and un-shared co-regulated interactions, i.e., more control sentences and more complex speech during un-shared co-regulated patterns.

## Materials and Methods

### Participants

One hundred and one mothers and their 3 month-old (corrected age for PT) infants (PT = 56 and FT = 55) participated in the study. Among those, 14 mothers of PT infants and 12 mothers of FT infants were excluded due to failure to complete study questionnaires (i.e., Parenting Stress Index). The final sample consisted of 86 dyads (PT = 42 and FT = 44).

Preterm infants born <37 weeks gestational age were included. Exclusion criteria for both preterm and full-term groups were the presence of genetic abnormalities, severe neurodevelopmental impairment, and/or neurosensory disabilities (e.g., blindness or deafness).

Most mothers (mean age: PT = 33.63, SD = 5.05; FT = 35.23, SD = 4.84) had a middle or high school level of education: 38.8% had a high school degree; 55.3% graduated college or had a master’s degree; 5.9% had less than a high school education. Preterm infants (43% Males; 78% First born) and FT infants (59% Males; 66% First born) had a mean gestational age of 30.71 (SD = 2.63) and 39.41 (SD = 1.18) weeks, and a mean birth weight of 1,379 (SD = 437.92) and 3,397 (SD = 406.97) grams, respectively. All infants were singletons.

### Procedure

Mothers were invited to participate with their infant in a videotaped observational session when their infant was 3 months old (for preterm infants the corrected age was used). FT dyads were recruited via public services and advertisements, PT dyads were recruited by nurses and doctors of the hospital where they were born. All mothers completed and signed a consensus form before participation. The session consisted of 3 min of face-to-face interaction with the infant seated on an infant seat, and the mother seated directly in front of the infant, facing a mirror which was located behind the infant’s seat so that both partners’ faces could be clearly seen. After a brief introduction, mothers were asked to interact with their infants, as they would do at home.

After the interactive episode, mothers were asked to complete the short version of the Parenting Stress Index. Each session was entirely transcribed according to the CHAT transcription system (Codes for the Human Analysis of Transcripts) of the CHILDES computational system (Child Language Data Exchange System) (MacWhinney, 2000).

The study was approved by the Ethical Committee of the Department.

### Coding and Measures

#### Dyadic Co-regulation

Mother–infant interactions were coded using the Revised Relational Coding System (Fogel et al., 2003) to capture the quality of the interactive involvement between mothers and infants. The whole interaction was coded. The quality of dyadic behaviors ranges from the absence of orientation of one partner to the other, to the mutual and continuous adjustment of their respective actions.

The coding system includes five global categories of communicative interactions: unilateral, asymmetrical, symmetrical, disruptive, and unengaged. In the present study we considered the global categories of *symmetrical co-regulation* (characterized by both partners adjusting their communicative actions to the continuously changing actions of the partner, and engaging in active, mutual engagement, and sharing experience via vocal and non-vocal behaviors) and *unilateral co-regulation* (characterized by only one partner trying to engage the other, while the other is absorbed in their own activity and failing to pay attention to the partner, or respond to the partner’s initiations).

The co-regulation patterns were coded every second from the videotapes by a trained coder, using the Mangold Interact 18 software. The relative total duration of each pattern was computed. An independently trained coder processed 25% of the sessions to compute inter-observer reliability. The Kappa values were 0.86 for symmetrical co-regulation and 0.94 for unilateral co-regulation.

#### Parenting Stress

Mothers were asked to complete the PSI-Short Form questionnaire (PSI-SF; (Abidin, 1995)). The PSI-SF is a commonly used questionnaire designed to measure stress in the parent-child system and to identify caregivers that are most in need of support. The PSI-SF includes 36 items rated from 1 to 5 on a Likert scale (1 = strongly disagree; 5 = strongly agree), and consists of three subscales, of 12 items each: Parental Distress (PSI-PD), Parent-Child Dysfunctional Interaction (PSI-P-CDI) and Difficult Child (PSI-DC). High values indicate more parenting stress. For the present study, the Parental Distress subscale was used (Cronbach’s α: PD = 0.86). This scale explores the stress related to the parent’s perception of her/his child-rearing competences, the level of spousal conflicts or support, and the restrictions placed by parental role. Item mean scores were calculated by dividing the sum of item scores by the number of items comprising that scale.

#### Infant-Directed Speech: Linguistic Features

Maternal vocal productions were coded in order to analyze:

- Verbosity: Rate per minute of utterances, word types and tokens (Phillips, 1973; Henning et al., 2005).
- Lexical variability: Type/token ratio (*TTR*), which is a ratio of the number of types to tokens (Johnson, 1944; Broen, 1972; Phillips, 1973).
- Syntactic complexity: *MLU*, which is a ratio of the total number of words spoken to the total number of utterances (Snow, 2009).

#### Infant-Directed Speech: Pragmatic Features

In order to classify the pragmatic meaning of maternal productions, these were divided into the following categories and the percentages for each category over the total number of utterances considered were calculated (Longobardi, 1992):

- Conversational: Sentences used to promote and maintain the conversation with the infant (i.e., emphatic sentences and comments “You look happy,” open questions “What are you looking at?”, comments on the present/past activity of the infant “We are playing together”).
- Control: Sentences used to re-orient infant attention, to direct infant attention toward something (i.e., direct requests “Speak to me,” claiming infant attention “Ehy, look at me”).
- Preverbal: Sounds and sentences using typical baby-talk words, repetition of infant’s sounds.

### Analyses Plan

We first computed descriptive statistics and bivariate correlations among study variables in the full sample and separately in the two groups. The two birth groups were compared for mean values along the investigated variables. Afterward, to explore the single and additive role of birth status, dyadic co-regulation and parenting stress, and the interplay between dyadic co-regulation and parenting stress with birth status on IDS characteristics, we estimated and compared several multivariate models and then explored parameters of the best selected model More specifically, pertaining to predictors, first birth status was included in the model. Then, we considered the additive role of the psychological variables investigated (co-regulation and parenting stress). Finally, we included the interaction term between birth status and each of the dyadic/maternal variables considered to see if co-regulation and parenting stress differently predicted IDS depending on birth status. The Akaike Information Criterion (AIC) was used for model comparison, with lower values providing more support to a model against the others. The first group of multivariate models included as outcome variables IDS linguistic characteristics (verbosity, TTR and MLU), and then we considered IDS pragmatic characteristics (conversational, control and preverbal sentences) Regression parameters were explored for the best fitting model. Analyses were run using the statistical software R, using Lavaan package.

## Results

### Descriptive Statistics

Means, SDs, and correlation values among variables of interest in the full sample are reported in Table 1. Within the IDS features, we observed significant correlations as expected. Symmetrical co-regulation was negatively associated with unilateral co-regulation as expected (*r* = −0.56). Parenting stress was positively associated with TTR, and MLU (*r* = 0.28 and *r* = 0.22, respectively).

**TABLE 1:** Descriptive and bivariate correlations for the full sample.

		Mean (SD)	1	2	3	4	5	6	7	8	9
1	Verbosity	26.16 (8.52)	−								
2	TTR	0.40 (0.09)	−0.46*	−							
3	MLU	4.07 (0.94)	−0.19	0.10	−						
4	% Conversational	56.84 (15.36)	−0.19	0.01	0.46**	−					
5	% Control	30.89 (13.97)	0.25*	−0.22*	−0.30**	−0.68**	−				
6	% Preverbal	7.59 (10.77)	−0.08	0.25*	−0.33**	−0.42**	−0.30**	−			
7	Parenting stress	1.83 (0.56)	−0.16	0.28**	0.22*	0.08	−0.14	0.15	−		
8	Unilateral co-regulation	0.31 (0.26)	−0.09	0.21	−0.11	−0.09	0.19	−0.05	0.08	−	
9	Symmetrical co-regulation	0.21 (0.16)	0.18	−0.16	0.06	0.02	−0.18	0.15	0.03	−0.56**	−

*p > 0.05, **p < 0.01.

Exploration of bivariate associations among investigated variables run separately for the two birth status groups (see Table 2) suggested that IDS linguistic characteristics were strongly associated with Parenting Stress in the FT group, but not in the PT group. More stressed FT mothers were observed to speak less (verbosity: *r* = −0.37) and demonstrated higher lexical variability and syntactic complexity (TTR: *r* = 0.34, MLU: *r* = 0.36). Similarly, IDS pragmatic characteristics were significantly associated with Unilateral and Symmetrical co-regulation only in the FT group. Mothers of FT infants pronounced less conversational and more controlled sentences when the dyad spent more time in unilateral co-regulation (*r* = −0.34 and *r* = 0.52, respectively) and mothers used less control sentences when the dyad spent more time in symmetrical co-regulation (*r* = −0.44).

**TABLE 2:** Descriptive and bivariate correlations in the preterm (above the diagonal, *n* = 42) and full-term (below the diagonal, *n* = 44) groups.

		Mean (SD) PT	Mean (SD) FT	1	2	3	4	5	6	7	8	9
1	Verbosity	21.91 (6.32)	30.20 (8.43)	−	−0.43**	−0.01	−0.28	0.19	0.03	−0.10	−0.11	0.10
2	TTR	0.41 (0.09)	0.39 0.09)	−0.55**	−	−0.14	0.02	−0.19	0.33*	0.24	0.33*	−0.19
3	MLU	4.33 (0.91)	3.83 0.91)	−0.12	0.31*	−	0.47**	−0.43**	−0.17	0.18	−0.17	0.18
4	% Conversational	60.77 (14.13)	53.09 (15.69)	0.05	−0.03	0.39**	−	−0.77**	−0.29	0.17	0.10	−0.08
5	% Control	29.02 (13.47)	32.68 (14.35)	0.24	−0.24	−0.13	−0.59**	−	−0.22	−0.14	−0.08	0.06
6	% Preverbal	5.27 (6.67)	9.80 (13.30)	−0.31*	0.26	−0.37*	−0.45**	−0.41**	−	0.07	0.21	−0.13
7	Parenting Stress	1.75 (0.54)	1.90 (0.58)	−0.37*	0.34*	0.36*	0.08	−0.18	0.16	−	0.30	−0.06
8	Unilateral co-regulation	0.34 (0.28)	0.29 (0.23)	0.01	0.06	−0.11	−0.34*	0.52**	−0.17	−0.12	−	−0.54**
9	Symmetrical co-regulation	0.19 (0.17)	0.23 (0.16)	0.18	−0.13	0.01	0.18	−0.44**	0.27	0.08	−0.58**	−

*p > 0.05, **p < 0.01.

The One Way ANOVA evidenced only few differences between PT and FT groups. When speaking to their infants, mothers of PT infants, talked less [verbosity: *F*_(1_,_84)_ = 26.45, *p* < 0.001], demonstrated higher syntactic complexity [MLU: *F*_(1_,_84)_ = 6.37, *p* = 0.01], more conversational and less preverbal sentences [*F*_(1_,_84)_ = 5.67, *p* = 0.02 and *F*_(1_,_84)_ = 3.91, *p* = 0.05, respectively] than FT mothers. Concerning parenting stress [*F*_(1_,_84)_ = 0.49, *p* = 0.21] and dyadic co-regulation [unilateral: *F*_(1_,_84)_ = 0.78, *p* = 0.38; symmetrical: *F*_(1_,_84)_ = 1.27, *p* = 0.26], no differences emerged between the groups.

### Multivariate Regression Models

#### Birth Status and Unilateral Co-regulation on Infant-Directed Speech Linguistic Characteristics

Comparison of multivariate regression models (see Table 3) demonstrated that model 2 (which included birth status and dyadic unilateral co-regulation as single effects) outperformed the other models. Standardized estimates of model 2 are reported in Table 4. Only regression parameters of the effect of birth status were significant at *p* < 0.05, except for the role of birth status on TTR. Results showed that mothers of PT infants spoke less (verbosity: β = 0.97, *p* < *0.001*), and with lower syntactic complexity (MLU: β = −0.52, *p* = *0.007*) than mothers of FT infants. None of the parameters regarding the effect of unilateral co-regulation were significant.

**TABLE 3:** Model comparison, effects of birth status, and dyadic co-regulation: AIC.

	IDS linguistic	IDS pragmatic

Model	AIC	AIC
Model 1: Birth status	262.51	1876.5
Model 2: Birth status, unilateral co-regulation	**262.29**	1876.3
Model 3: Birth status, unilateral co-regulation, birth status × unilateral co-regulation	266.98	**1868.5**
Model 1: Birth status	**262.51**	1876.5
Model 2: Birth status, symmetrical co-regulation	264.63	1877.5
Model 3: Birth status, symmetrical co-regulation, birth status × symmetrical co-regulation	269.31	**1874.3**

*In bold are highlighted models receiving more support for each set of outcome variables considered (IDS linguistic and IDS pragmatic).*

**TABLE 4:** Multivariate analysis on IDS linguistic and pragmatic characteristics: Standardized estimated parameters of models 2 and 3, respectively.

	Verbosity	*TTR*	MLU	Conversational	Control	Preverbal
	β *(p)*	β *(p)*	β *(p)*	β *(p)*	β *(p)*	β *(p)*
Birth status	0.97 (<0.001)	−0.01 (0.712)	−0.52 (0.007)	0.67 (0.889)	−6.85 (0.113)	8.98 (0.011)
Unilateral co-regulation	−0.15 (0.682)	0.07 (0.05)	−0.49 (0.189)	33.33 (0.068)	−39.68 (0.015)	19.55 (0.139)
Birth status × Unilateral co-regulation	−	−	−	−28.20 (0.021)	35.95 (0.001)	−14.68 (0.096)

#### Birth Status and Unilateral Co-regulation on Infant-Directed Speech Pragmatic Characteristics

**FIGURE 1:**
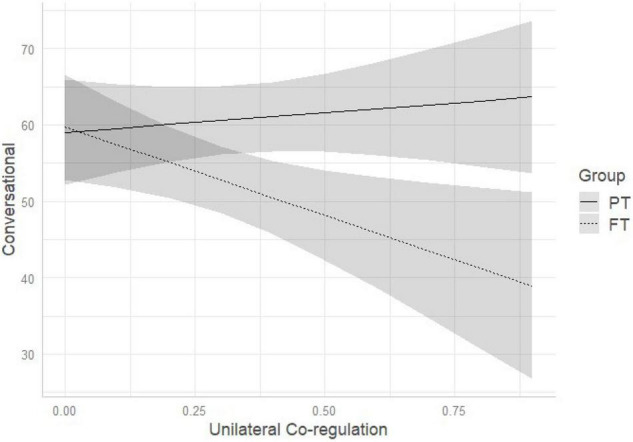
Interaction among birth status and unilateral co-regulation on IDS conversational pragmatic sentences. PT, preterm; FT, full-term.

**FIGURE 2:**
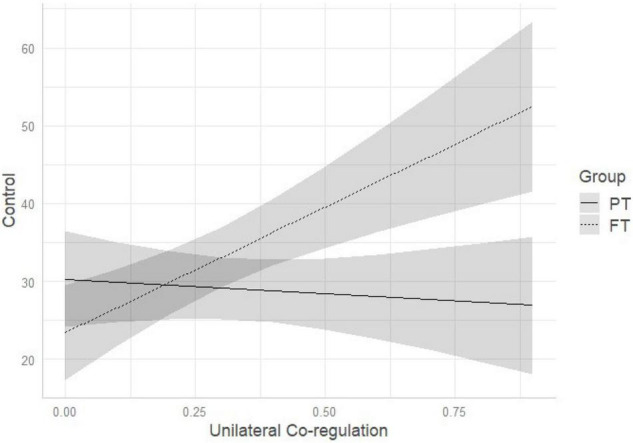
Interaction among birth status and unilateral co-regulation on IDS control pragmatic sentences. PT, preterm; FT, full-term.

Comparison of multivariate regression models (see Table 3) demonstrated that model 3 (which included birth status, dyadic unilateral co-regulation and their interaction) outperformed the other models. Standardized estimates of model 3 are reported in Table 4. Regression parameters of the interaction effect were significant at *p* < 0.05 for conversational (β = −28.20, *p* = 0.021) and control sentences (β = 35.95, *p* = 0.001) (see Table 4). As represented in Figure 1, the more time the dyad spends in unilateral co-regulation, the more likely FT mothers are to reduce the quantity of conversational sentences while PT mothers continue using a high percentage of conversational sentences. Conversely, the more time the dyad spends time in unilateral co-regulation, the more likely FT mothers are to use control sentences, while PT mothers do not vary in the amount of control sentences pronounced (see Figure 2).

#### Birth Status and Symmetrical Co-regulation on Infant-Directed Speech Linguistic Characteristics

Comparison of multivariate regression models (see Table 3) demonstrated that model 1 (which included only birth status as a single effect) outperformed the other models. Standardized estimates of model 1 are reported in Table 5. Regression parameters of the effect of birth status were significant at *p* < 0.05, except for the role of birth status on TTR. Results showed that mothers of PT infants spoke less (verbosity: β = 0.97, *p* < 0.001), and with lower syntactic complexity (MLU: β = −0.50, *p* = 0.011) compared with mothers of FT infants. None of the parameters regarding the effect of symmetrical co-regulation were significant.

**TABLE 5:** Multivariate analysis on IDS linguistic and pragmatic characteristics: Standardized estimated parameters of models 1 and 3, respectively.

	Verbosity	*TTR*	MLU	Conversational	Control	Preverbal
	β *(p)*	β *(p)*	β *(p)*	β *(p)*	β *(p)*	β *(p)*
Birth status	0.97 (<0.001)	−0.01 (0.586)	−0.50 (0.011)	−12.87 (0.013)	13.67 (0.003)	−1.52 (0.672)
Symmetrical co-regulation	−	−	−	−30.36 (0.319)	49.33 (0.068)	−32.64 (0.123)
Birth status × Symmetrical co-regulation	−	−	−	23.91 (0.220)	−44.74 (0.010)	27.44 (0.042)

#### Birth Status and Symmetrical Co-regulation on Infant-Directed Speech Pragmatic Characteristics

**FIGURE 3:**
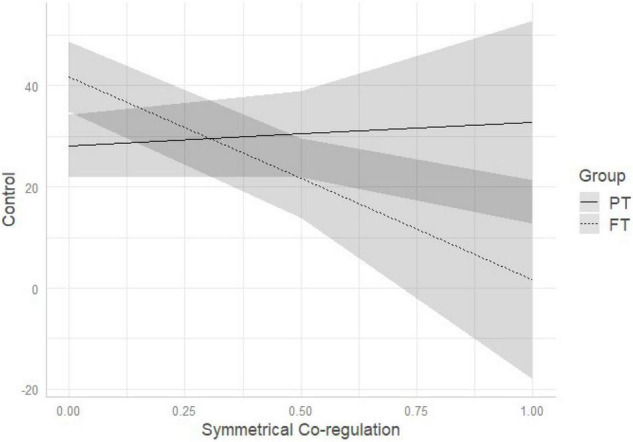
Interaction among birth status and symmetrical co-regulation on IDS control pragmatic sentences. PT, preterm, FT, full-term.

**FIGURE 4:**
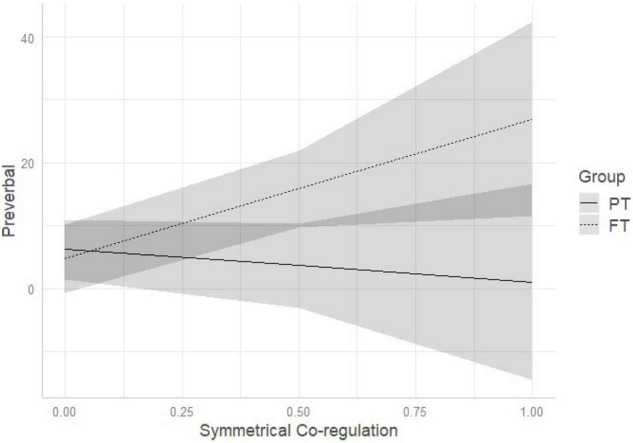
Interaction among birth status and symmetrical co-regulation on IDS preverbal pragmatic sentences. PT, preterm, FT, full-term.

Comparison of multivariate regression models (see Table 3) demonstrated that model 3 (which included birth status, dyadic symmetrical co-regulation and their interaction) outperformed the other models. Standardized estimates of model 3 are reported in Table 5. Regression parameters of the interaction effect were significant at *p* < 0.05 for control (β = −44.74, *p* = 0.010) and preverbal sentences (β = 27.44, *p* = 0.042) (see Table 5). As represented on Figure 3, the more time the dyad spends time in symmetrical co-regulation, the less FT mothers use control sentences, while PT mothers do not vary in the amount of use of control sentences. Conversely, the more time the dyad spends time in symmetrical co-regulation, the more likely FT mothers are to increase the quantity of preverbal sentences, while PT mothers continue using a low percentage of preverbal sentences (see Figure 4).

#### Birth Status and Parenting Stress on Infant-Directed Speech Linguistic Characteristics

Comparison of multivariate regression models (see Table 6) showed model 2 (which included birth status and parenting stress as single additive effects) outperformed the other models. Standardized estimates of model 2 are reported in Table 7. All regression parameters were significant at *p* < 0.05 except for the role of birth status on TTR. Results showed that mothers of PT infants spoke less (verbosity: β = 1.03, *p* < 0.001), and demonstrated lower syntactic complexity (MLU: β = −0.56, *p* = 0.003) than mothers of FT infants. Moreover, for both PT and FT groups, mothers with higher levels of parenting stress spoke less (verbosity: β = −0.40, *p* = 0.012), and demonstrated higher lexical variability (TTR: β = 0.045, *p* = 0.005) and higher syntactic complexity (MLU: β = 0.443, *p* = 0.008).

**TABLE 6:** Model comparison, effects of birth status and parenting stress: AIC.

	IDS linguistic	IDS pragmatic

Model	AIC	AIC
Model 1: Birth status	262.51	**1876.5**
Model 2: Birth status, parenting stress	**253.31**	1877.4
Model 3: Birth status, parenting stress, birth status × parenting stress	256.26	1882.9

*In bold are highlighted models receiving more support for each set of outcome variables considered (IDS linguistic and IDS pragmatic).*

**TABLE 7:** Multivariate analysis on IDS linguistic and pragmatic characteristics: Standardized estimated parameters of models 2 and 1, respectively.

	Verbosity	*TTR*	MLU	Conversational	Control	Preverbal
	β *(p)*	β *(p)*	β *(p)*	β *(p)*	β *(p)*	β *(p)*
Birth status	1.03 (<0.001)	−0.02 (0.346)	−0.56 (0.003)	−7.68 (0.016)	3.66 (0.217)	4.52 (0.045)
Parenting stress	−0.40 (0.012)	0.04 (0.005)	0.44 (0.008)	−	−	−

#### Birth Status and Parenting Stress on Infant-Directed Speech Pragmatic Characteristics

Comparison of multivariate regression models (see Table 6) demonstrated that model 1 (which included birth status as single effect) outperformed the other models. Standardized estimates of model 1 are reported in Table 7. All regression parameters were significant at *p* < 0.05 except for the role of birth status on control sentences. Results showed that for mothers of PT infants, IDS was characterized by more conversational (β = −7.68, *p* = 0.016) and less preverbal (β = 4.52, *p* = 0.045) sentences compared with mothers of FT infants.

## Discussion

The present study aimed to explore the intra- and inter-individual differences of the linguistic and pragmatic features of IDS, directed to 3-month-old full-term and preterm infants. We found evidence of interactive effects between individual (infant and maternal) and dyadic factors, on IDS characteristics. Recognizing that birth status (i.e., preterm birth) is an important condition affecting the infant, mother and dyad (Spinelli et al., 2013, 2016; Poehlmann-Tynan et al., 2015), we chose to examine patterns of IDS of mothers of PT and FT infants, and their associated predictors. It is well described that in the first year of life, PT infants manifest interactive difficulties, especially related to the communicative and regulatory aspects of infant’s interaction with the environment (Goldberg and DiVitto, 1995; Sajaniemi et al., 1998). For this reason, mothers of PT infants are expected to adapt their interactive style so as not to over- or under- stimulate the infant (Feldman and Eidelman, 2007). Our findings regarding group differences evidenced that, during face-to-face interactions, mothers of PT infants spoke less, and vocalized with higher syntactic complexity, with the use of more conversational sentences and less preverbal sentences than mothers of FT infants. The linguistic and pragmatic features of IDS in mothers of PT infants are suggestive of a more complex pattern of IDS, evidenced by a less-talkative interactive style. This syntactic complexity is typically manifest in IDS directed to older infants, because this pattern of communication is more difficult to follow for a younger infant (Suttora and Salerni, 2011; Genovese et al., 2020). At 3 months, when infants have a limited ability to follow conversational exchanges, more complex sentences might result in less proto-conversational dyadic exchanges because the infant has less opportunities to vocalize in response to the sound of maternal voice. We have some possible explanations for these findings. Regarding PT dyads, one possibility is that this interactive style is related to the PT infant’s communicative difficulties, resulting in a lower responsiveness to the mother’s vocalizations. When experiencing less feedback from their PT infant, these mothers might therefore speak in a more complex way, because they don’t expect a consistent participation of the infant during their vocal exchanges. At the same time, they leave more silent moments in which the infant has the space he/she need to respond to IDS stimulation. To better examine this possibility, future studies should consider the reciprocal influence of PT infants vocal and interactive responses to maternal IDS.

Regarding the role of dyadic co-regulation and IDS in PT versus FT infants, we found no differences between the groups concerning the quality of the dyadic, symmetrical and unilateral, co-regulation. Expressed differently, our results suggest that different patterns of IDS (between FT and PT) are not associated with fewer moments of co-regulated attention and affect. This is relevant because many studies considered the interactive qualities of PT mothers to be under- or over-stimulating, which was presumed to be suboptimal compared to what was observed in typical FT mothers (Forcada-Guex et al., 2006). However, the lack of differences in co-regulation between FT and PT groups suggests that the interactive vocal communication of PT mothers may be just as effective in contributing to the creation of dyadic shared moments. We hypothesize that this pattern of maternal vocalizations is a part of a specific interactive style that is attuned to the communicative and interactive abilities of PT infants, although this is an area in need of further research. While most studies have focused on difference between PT and FT dyads, with the aim to evaluate the adequacy with FT dyads as comparisons, future research should focus on describing the specific characteristics of PT dyads as probably mothers’ adaptations to the specificities of premature birth condition, and on exploring within PT dyads differences in associations with later child development outcomes (Poehlmann-Tynan et al., 2015).

One notable difference emerged between the groups when exploring how mothers vary their IDS with respect to the time spent in co-regulated interaction. We observed that the duration of shared versus un-shared moments was associated with different pragmatic features of IDS in FT, but not PT, mothers. In FT dyads, the greater time spent in moments of un-shared attention and affect (i.e., unilateral co-regulation) was associated with an IDS characterized by reduced conversational and increased control sentences. Conversely, the greater time spent in shared moments of attention and affect (i.e., symmetrical co-regulation) was associated with increased use of preverbal, and decreased use of control sentences. Conversational sentences have, as its primary purpose, to keep open the communicative channels between two individuals when they are engaged in the same subject or are having a shared emotional experience. This is manifest by, for example, making comments, offering compliments, and asking open-ended questions. In contrast, control sentences are used to redirect, modify, and capture the attention of another when the other individual is focused on something different. This is manifested by, for example, calling or giving orders (Longobardi, 1992). Consistently, FT mothers who lose more the attention of their infants, reduce more the quantity of comments and open questions, and use a conversational style to try to elicit the infant’s attention again. On the other hand, when experiencing more moments of co-regulated attention and affect, FT mothers tend to use fewer control sentences and more preverbal sentences, with IDS characterized by repeating the infant’s vocalizations, singing, or making animal sounds. What we observed is that FT mothers demonstrate the ability to adapt their IDS to the quality of dyadic co-regulation, whereas this adaptability is not present in PT mothers. Of note, the pragmatic features of PT mothers’ IDS did not vary according to dyadic co-regulation. One possible explanation for this finding is that PT mothers are less flexible in using verbal communication as an interactive modality to elicit or maintain infant attention, and preferentially use other interactive modalities (e.g., touch) instead (Wigley et al., 2022). This may be partially attributed to the more ambiguous and less frequent vocal feedback received from the infant. Before labeling this lack of variation of IDS as suboptimal, it would be helpful to observe whether this pattern of interaction is observed at other ages, when infants are expected to be more vocally interactive, and whether there are associations with later infant development outcomes. Further qualitative investigation may be needed on the comparison between PT and FT dyadic communication to better describe their specificities. Moreover, moment by moment analyses of IDS features as well as sequential analyses would help understanding how mothers adapt their IDS over time and according to changes in the quality of co-regulation patterns.

Consistent with previous studies, we did not find differences in parenting stress between the two groups (Gray et al., 2012; Suttora et al., 2020b). The experience of a preterm delivery, even if it was potentially traumatic for mothers, did not result in higher self-reported parenting stress in mothers of PT infants compared to mothers of FT infants at 3 months of age (Gray et al., 2012). When exploring the effect of parenting stress and birth status on IDS, the multivariate models demonstrated no interactive effects of stress with birth status, suggesting that the perceived parenting stress has similar effects in FT and PT groups. In both FT and PT groups, mothers who reported perceiving their parenting role as a stressful experience, demonstrated lower verbosity, higher lexical variability, and higher syntactic complexity. This less simple IDS is more typical of conversations directed to adults or to older children and might be considered a lower ability to connect and attune to the infant needs and communicative abilities (Genovese et al., 2020). Parenting stress might therefore be considered a maternal wellbeing risk factor which affects the quality of linguistic input. Long-term consequences of this effect should be examined in future studies. Since the linguistic characteristics of IDS have been associated with infants’ and children’s language development (Soderstrom, 2007), this raises the possibility that higher levels of parenting stress may also reduce the positive impact of IDS on language development.

We would like to acknowledge some limitations of the current study. First, while our results were relevant, our sample sizes were not big and we included only 3-month-old infants. A larger sample followed longitudinally would have allowed us to explore additional interactive effects. An additional limitation is that our PT sample was quite homogeneous, composed of low-risk PT infants and of well educated low-risk mothers. Additional research should examine these associations in a more at-risk population of preterms to identify differential effects both at maternal, infant, and dyadic levels. With a larger and more at-risk sample, also the associations of IDS with the other co-regulation patterns, i.e., asymmetrical and unengaged, could be explored. Further studies should also consider the paternal dyadic communication and explore if these findings are replicable in father-infant dyads. Lastly, we did not explore infant vocal communication during the interaction. This information, as well as the inclusion of non-verbal infant and maternal cues, would help interpret our findings and should be the focus of future studies.

Despite these limits, this study presents several strengths. To the best of our knowledge, this is the first study to examine infant, maternal, and dyadic factors and their associations with characteristics of IDS. This study highlights the need to go beyond exploring IDS effects on language development, and to consider its potential importance when exploring the quality of dyadic interaction and its role in sharing attention, affect and meaning between the mother and the infant (Saint-Georges et al., 2013). Our results could be useful in structuring interventions aimed to promote PT dyads quality of interaction. Knowing the specific characteristic of PT mothers’ vocal communication could help defining more appropriated and well-designed interventions by helping mothers adapt their IDS to the specificities of infant communicative abilities in order to promote positive linguistic, attentive and affective outcomes.

## Data Availability Statement

The raw data supporting the conclusions of this article will be made available by the authors, without undue reservation.

## Ethics Statement

The studies involving human participants were reviewed and approved by the Ethics Committee of the Department of Neuroscience, Imaging and Clinical Sciences of the University G. d’Annunzio Chieti-Pescara. Written informed consent to participate in this study was provided by the participants’ legal guardian/next of kin.

## Author Contributions

MS and MF conceptualized the study and organized the data collection. MS and FL wrote the first draft of the manuscript. FL ran the analyses and wrote the results section. MG, ML, and SP conducted the data collection and coding. SD and PC coordinated preterm data collection. MF and PS revised the manuscript. All authors contributed to revision of the final version of the manuscript.

## Conflict of Interest

The authors declare that the research was conducted in the absence of any commercial or financial relationships that could be construed as a potential conflict of interest.

## Publisher’s Note

All claims expressed in this article are solely those of the authors and do not necessarily represent those of their affiliated organizations, or those of the publisher, the editors and the reviewers. Any product that may be evaluated in this article, or claim that may be made by its manufacturer, is not guaranteed or endorsed by the publisher.
